# Quantitative Predictions of Peptide Binding to Any HLA-DR Molecule of Known Sequence: NetMHCIIpan

**DOI:** 10.1371/journal.pcbi.1000107

**Published:** 2008-07-04

**Authors:** Morten Nielsen, Claus Lundegaard, Thomas Blicher, Bjoern Peters, Alessandro Sette, Sune Justesen, Søren Buus, Ole Lund

**Affiliations:** 1 Center for Biological Sequence Analysis, Department of Systems Biology, Technical University of Denmark, Lyngby, Denmark; 2 La Jolla Institute for Allergy and Immunology, San Diego, California, United States of America; 3 Laboratory of Experimental Immunology, Faculty of Health Sciences, University of Copenhagen, Denmark; National Cancer Institute, United States of America, Tel Aviv University, Israel

## Abstract

CD4 positive T helper cells control many aspects of specific immunity. These cells are specific for peptides derived from protein antigens and presented by molecules of the extremely polymorphic major histocompatibility complex (MHC) class II system. The identification of peptides that bind to MHC class II molecules is therefore of pivotal importance for rational discovery of immune epitopes. HLA-DR is a prominent example of a human MHC class II. Here, we present a method, NetMHCIIpan, that allows for pan-specific predictions of peptide binding to any HLA-DR molecule of known sequence. The method is derived from a large compilation of quantitative HLA-DR binding events covering 14 of the more than 500 known HLA-DR alleles. Taking both peptide and HLA sequence information into account, the method can generalize and predict peptide binding also for HLA-DR molecules where experimental data is absent. Validation of the method includes identification of endogenously derived HLA class II ligands, cross-validation, leave-one-molecule-out, and binding motif identification for hitherto uncharacterized HLA-DR molecules. The validation shows that the method can successfully predict binding for HLA-DR molecules—even in the absence of specific data for the particular molecule in question. Moreover, when compared to TEPITOPE, currently the only other publicly available prediction method aiming at providing broad HLA-DR allelic coverage, NetMHCIIpan performs equivalently for alleles included in the training of TEPITOPE while outperforming TEPITOPE on novel alleles. We propose that the method can be used to identify those hitherto uncharacterized alleles, which should be addressed experimentally in future updates of the method to cover the polymorphism of HLA-DR most efficiently. We thus conclude that the presented method meets the challenge of keeping up with the MHC polymorphism discovery rate and that it can be used to sample the MHC “space,” enabling a highly efficient iterative process for improving MHC class II binding predictions.

## Author Summary

CD4 positive T helper cells provide essential help for stimulation of both cellular and humoral immune reactions. T helper cells recognize peptides presented by molecules of the major histocompatibility complex (MHC) class II system. HLA-DR is a prominent example of a human MHC class II locus. The HLA molecules are extremely polymorphic, and more than 500 different HLA-DR protein sequences are known today. Each HLA-DR molecule potentially binds a unique set of antigenic peptides, and experimental characterization of the binding specificity for each molecule would be an immense and highly costly task. Only a very limited set of MHC molecules has been characterized experimentally. We have demonstrated earlier that it is possible to derive accurate predictions for MHC class I proteins by interpolating information from neighboring molecules. It is not straightforward to take a similar approach to derive pan-specific HLA-DR class II predictions because the HLA class II molecules can bind peptides of very different lengths. Here, we nonetheless show that this is indeed possible. We develop an HLA-DR pan-specific method that allows for prediction of binding to any HLA-DR molecule of known sequence—even in the absence of specific data for the particular molecule in question.

## Introduction

Major histocompatibility complex (MHC) molecules play an essential role in the host-pathogen interactions determining the onset and outcome of many host immune responses. While peptides derived from foreign, intracellular proteins and presented in complex with MHC class I molecules can trigger a response from cytotoxic T lymphocytes (CTL), MHC class II molecules present peptides derived from proteins taken up from the extra-cellular environment. They stimulate cellular and humoral immunity against pathogenic microorganisms through the actions of helper T lymphocytes. Only a small fraction of the possible peptides that can be generated from proteins of pathogenic organisms actually generate an immune response. In order for a peptide to stimulate a helper T lymphocyte response, it must bind MHC II in the endocytic organelles [1].

MHC molecules are extremely polymorphic. The number of identified human MHC (HLA) molecules has surpassed 1500 for class I and many thousands for class II [2]. This high degree of polymorphism constitutes a challenge for T cell epitope discovery, since each of these molecules potentially has a unique binding specificity, and hence a unique preference for which peptides to present to the immune system. Even though many of the alleles could be functionally very similar (i.e. have binding pockets that are similar to other alleles) it is often very difficult a priori to identify such similarities since subtle differences in binding pocket amino acids can lead to dramatic changes in binding specificity [3].

During the last decades, prediction of T cell epitopes has reached a level of accuracy which makes prediction algorithms a natural and integral part of most major large scale rational epitope discovery projects [4]–[6]. The single most selective event defining T cell epitopes is the binding of peptide fragments to the MHC complexes [7],[8]. However, most efforts in developing accurate prediction algorithms for MHC/peptide binding has focused on MHC class I (for review see [9]). Here, large-scale epitope discovery projects integrating high-throughput immunoassays [10] with bioinformatics has achieved highly accurate prediction algorithms covering large proportions of the human MHC class I allelic polymorphism [3],[11],[12]. The situation for MHC class II is quite different. Here, most prediction algorithms have been developed from small data sets covering a single or a few different MHC molecules [13]–[24]. Very limited work has been done on deriving HLA class II prediction algorithms with broad allelic coverage. To our knowledge, only three such publicly available method exists: Propred [25], ARB [17], and NetMHCII [26]. Propred is a publicly available version of the TEPITOPE method [27], which is an experimentally derived virtual matrix-based prediction method that covers 50 different HLA-DR alleles, and relies on the approximation that the peptide binding specificity can be determined solely from alignment of MHC pockets amino acids. NetMHCII and ARB are weight matrix data-driven methods derived from quantitative peptide/MHC binding data and covers 14 HLA-DR alleles (as well as some mouse MHC class II alleles). Most other HLA class II prediction methods have been trained and evaluated on very limited data sets covering only a single or a few different HLA class II alleles [13]–[23].

We have previously shown that a minimum number of 100–200 peptides with characterized binding affinity is needed to derive an accurate description of the binding motif for MHC class II alleles [26]. Characterizing the binding preference of each MHC molecule would therefore be an immense and very costly undertaking. In a recent paper, we have demonstrated that is a possible to derive accurate predictions for any HLA class I A and B loci protein of known sequence, by interpolating information from neighboring HLA class I molecules which have been experimentally addressed [3]. It would therefore seem natural to attempt as similar approach to derive a pan-specific HLA class II prediction algorithm. For two major reasons, however, the situation for HLA class II is very different from HLA class I. Firstly, quantitative binding data is only available for a few HLA class II alleles (only 14 HLA-DR alleles are characterized by more than 100 quantitative binding data points, the IEDB database November 2007, [28]). Secondly, the HLA class II binding groove is open at both ends allowing binding of peptides extended beyond the nonamer-binding core [29],[30]. A prerequisite for deriving a pan-specific binding prediction algorithm is therefore a precise alignment of the peptide-binding core to the HLA binding cleft. This alignment is essential since the algorithm underlying the pan-specific binding predictions relies on the ability to capture general features of the relationship between peptides and HLA sequences and interpret these in terms of a binding affinity. Such relationships can only by captured if the peptide is correctly aligned relative to the residues in the HLA binding cleft. We have recently published a method [26] for prediction of peptide-MHC class II binding that covers the 14 HLA-DR alleles which are populated with large amounts of quantitative peptide data in the IEDB database. This method provides a predicted binding affinity value for each peptide, together with an identification of the peptide-binding core, and it is based upon these predictions, we have developed this HLA-DR pan-specific method following the strategy described in [3].

In this work, we demonstrate how a pan-specific HLA-DR prediction method exploiting both peptide and primary HLA sequence can be used to accurately predict quantitative binding predictions for all HLA-DR molecules of known protein sequence. In particular, the method is capable of predicting the specificity of HLA-DR molecules with previously uncharacterized binding specificities thus demonstrating the true pan-specific nature of the method. The method and the benchmark data sets are available at http://www.cbs.dtu.dk/services/NetMHCIIpan.

## Results

**Figure 1:**
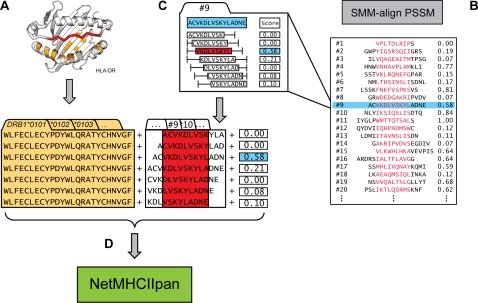
Schematic Illustration of the *NetMHCIIpan* Method. (A) The HLA-DR pseudo sequence is constructed from polymorphic HLA-DR residues in potential contact with a bound peptide. (B) Position specific scoring matrix (PSSM) and peptide core alignment (shown in red) is made for each allele using the SMM-align method [26]. N and C terminal peptide flanking regions, PFR, are identified as the up to three amino acids flanking the peptide-binding core. (C) Suboptimal peptides are presented to the *NetMHCpan* method with binding values normalized to the optimal peptide score (for the peptide shown in red) as described in Materials and Methods. (D) The *NetMHCIIpan* method is trained integrating data from all alleles. Input to the artificial neural network training includes the peptide core, composition and length of the N and C terminal PFR, length of the source peptide as well as the normalized binding affinity value (for details see Materials and Methods).

We trained the pan-specific HLA-DR prediction method as schematically illustrated in Figure 1. Both peptide sequences and HLA primary sequence information were used as input to the method. The peptide core and peptide flanking residues (PFR) were identified using the stabilized matrix alignment method [26]. Multiple register peptides were presented to the method in terms of the normalized measured binding affinity as illustrated in Figure 1B. By including both the peptide and HLA primary sequence, the pan-specific method is able to predict binding of peptides to all HLA-DR molecules even in the absence of data characterizing its binding specificity.

### Leave-One-Out Validation

To validate the pan-specific method, we conducted a leave-one-molecule out (LOO) experiment covering all 14 HLA-DR alleles included in the IEDB data set. For each allele, an artificial neural network (ANN) pan-specific predictor was trained as described in Materials and Method using all peptide data from the IEDB data set except the data for the HLA-DR molecule in question. Next, peptide binding affinity values for the HLA-DR molecule in question were obtained as the ANN prediction score for the optimal nonamer peptide core. The experiment thus simulates prediction of binding to hitherto un-characterized HLA-DR molecules. The predictive performance for each HLA allele was measured in terms of the AUC value [31] and Pearson's correlation [32]. Values for the Spearman's rank correlation [32] are given in Table S1. For each allele, we compared the LOO performance to that of the TEPITOPE method [27] for the alleles covered by this method, and a conventional single allele predictor (SMM-align [26]) trained on data from the most closely related HLA molecule as identified by similarity between the HLA sequences (Neighbor).

The results shown in Table 1 clearly demonstrate the predictive power of the pan-specific LOO method. The LOO approach achieves the highest predictive performance for all 11 alleles covered by TEPITOPE, and only for two alleles (DRB1*1302, and DRB4*0101) is the performance of the single allele neighbor method (SMM-align) better than that of the pan-specific LOO method. These differences are statistically significant (*p*<0.001 and *p* = 0.001, respectively, Binomial test).

**Table 1:** Leave-One-Molecule-Out Benchmark Results in Terms of the AUC and Pearson's Correlation Values.

		AUC			Pearson		Neighbor	
Allele	N	LOO	Neighbor	TEPITOPE	LOO	Neighbor	Dist	Allele
DRB1*0101	5166	**0.778**	0.736	0.720	**0.570**	0.489	0.352	DRB1*0401
DRB1*0301	1020	**0.746**	0.679	0.664	**0.449**	0.337	0.277	DRB3*0101
DRB1*0401	1024	**0.775**	0.726	0.716	**0.598**	0.503	0.066	DRB1*0405
DRB1*0404	663	**0.852**	0.808	0.770	**0.684**	0.596	0.091	DRB1*0401
DRB1*0405	630	**0.808**	0.793	0.759	**0.597**	0.557	0.066	DRB1*0401
DRB1*0701	853	**0.825**	0.760	0.761	**0.655**	0.544	0.504	DRB1*0901
DRB1*0802	420	**0.841**	0.827	0.766	**0.631**	0.575	0.111	DRB1*1101
DRB1*0901	530	**0.653**	0.639		**0.388**	0.369	0.431	DRB5*0101
DRB1*1101	950	**0.799**	0.696	0.721	**0.588**	0.401	0.084	DRB1*1302
DRB1*1302	498	0.658	**0.675**	0.652	**0.351**	0.343	0.084	DRB1*1101
DRB1*1501	934	**0.738**	0.705	0.686	**0.535**	0.489	0.295	DRB1*0404
DRB3*0101	549	**0.716**	0.686		**0.444**	0.368	0.277	DRB1*0301
DRB4*0101	446	0.724	**0.726**		**0.469**	0.422	0.397	DRB1*0404
DRB5*0101	924	**0.831**	0.810	0.680	**0.633**	0.592	0.295	DRB1*1101
Ave*	14607	0.768	0.733		0.541	0.470		
Ave**		0.787	0.747	0.718				

The table gives the allele name, the number of peptides included in the IEDB data for each allele, the LOO, the nearest neighbor SMM-align [26] and TEPITOPE [27] performances, the later only for subset of alleles covered by that method. In bold is highlighted the highest performance for each allele. The Ave* and Ave** rows give the average performance over all 14 alleles, and over the 11 alleles covered by the TEPITOPE method, respectively.

**Figure 2:**
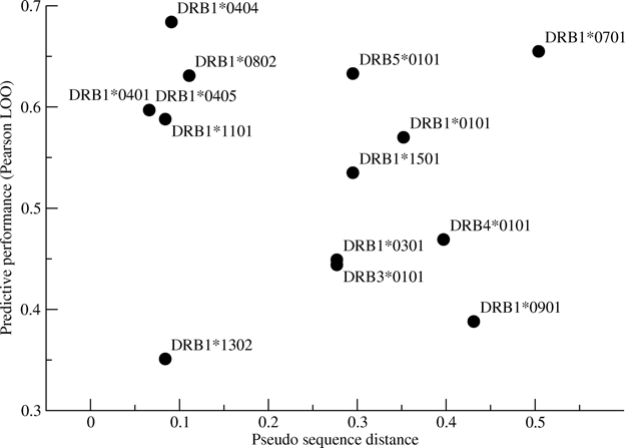
Predictive Performance in Terms of the Pearson's Correlation of the LOO Pan-Specific Method as a Function of the Distance to Its Nearest Neighbor HLA-DR Allele. The nearest neighbor distance is estimated as described in Materials and Methods.

The predictive performance of the pan-specific method relies on the ability to interpolate information from “neighboring” alleles in HLA specificity space and interpret this information in terms of binding affinities. It is thus expected that the pan-specific method should perform best in cases where closely related HLA molecules are included in the training of the method. The data in Table 1 and Figure 2 illustrates that this is indeed the case. Except for the two outliers DRB1*1302, and DRB1*0701 the plot shows the clear relation that alleles with close nearest neighbors tend to be predicted with a higher accuracy compared to alleles with large distances to their nearest neighbor.

### Cross-Validation

**Figure 3:**
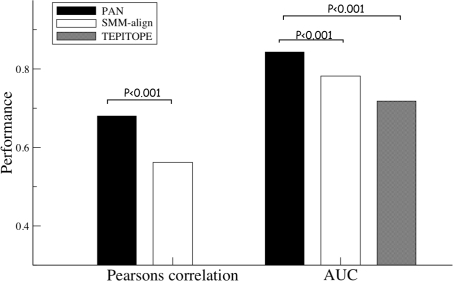
Cross-Validation Benchmark Evaluation. The predictive performance of the pan-specific, SMM-align, and TEPITOPE methods compared in terms of the Pearson's correlation and AUC values averaged over the 11 alleles covered by the TEPITOPE method, respectively (data for the individual alleles is given in Table S2).

Next, the final *NetMHCIIpan* method was trained on the complete datasets in a fivefold cross-validated manner abandoning the leave-one-out approach (see Materials and Methods). We compare the performance of the *NetMHCIIpan* method to that of a conventional single allele prediction method (SMM-align) and the TEPITOPE method in terms of both the AUC values and the Pearson's correlation coefficient (the latter is only included for the *NetMHCIIpan* and SMM-align methods, since the TEPITOPE method does not provide output values that are linearly related to the peptide binding affinity). The summary of this benchmark calculation is shown in Figure 3 (for details see Table S2).

The results show how the pan-specific method is capable of integrating information from neighboring HLA-DR molecules, and thus boosting the predictive performance beyond that of the conventional single allele methods like SMM-align and TEPITOPE. For all 14 alleles included in the benchmark, the pan-specific method outperforms the two other methods (*p*<0.001, Binominal test).

### Validation Using a Hitherto Uncharacterized HLA-DR Molecule

The ultimate validation of a pan-specific method for HLA-DR peptide binding predictions would be to identify which peptides that will bind to a hitherto un-characterized HLA-DR molecule. We therefore conducted such an experiment where a set of 256 15mer peptides were tested in an *in vitro* binding assay for binding to the HLA-DRB1*0813 molecule (described in Materials and Methods). Of the 20 top scoring peptides, 75% were shown to bind with a *K*_D_ values below 1000 nM, and 50% were shown to bind stronger than 50 nM. A performance summary of this experiment is shown in Table 2. This experiment demonstrates how the pan-specific prediction approach can identify peptide-binding motifs even in the absence of any data for the specific query HLA-DR molecule.

**Table 2:** Prospective Validation Using an Hitherto Uncharacterized HLA Molecule.

	AUC	Spearman's rank correlation	Pearson's correlation
Pan-specific	0.783	0.582	0.567
TEPITOPE	0.769	0.547	

Predictive performance values for the *Pan-specific* and TEPITOPE [27] methods, respectively, on a set of 256 15mer peptides. The AUC value (area under the operator receiver curve) was calculated using an IC50 binding threshold value of 500 nM. Note, that the Pearson's correlation is not informative for the TEPITOPE method since this method gives large negative (−999) scores to disfavored amino acids on certain positions.

### Identifying Endogenously Presented Peptides

**Figure 4:**
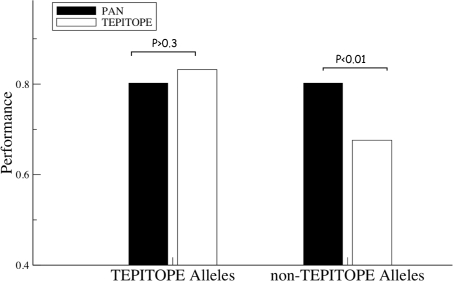
Prediction of Endogenously Presented Peptides. The benchmark data set consists of 584 HLA-DR restricted ligands covering 28 HLA-DR alleles downloaded from the SYFPEITHI database as described in the text. For alleles not covered by the TEPITOPE method, the closest allele covered by the TEPITOPE method as identified by sequence similarity between the HLA pseudo-sequences is used. TEPITOPE Alleles give the average AUC performance over the 17 alleles covered by the TEPITOPE method, and non-TEPITOPE Alleles give the average AUC performance over the 11 alleles *no*t covered by the TEPITOPE method (data for the individual alleles is given in Table S3).

The *NetMHCIIpan* method was further validated using a large set of data from the SYFPEITHI database [29], which were not included in the training data of the *NetMHCIIpan* method. This set consists of 584 HLA ligands restricted to 28 different HLA-DR alleles. For every peptide, the source protein was found in the SwissProt database [33]. If more than one source protein was possible, the longest protein was chosen. The source protein was split into overlapping peptide sequences of the length of the HLA ligand. All peptides except the annotated HLA ligand were taken as negative peptides. We are aware that this is a strong assumption, since suboptimal peptides that could be presented on the HLA molecule are counted as negatives. For each protein-HLA ligand pair the predictive performance was estimated as the AUC value. The summary of this benchmark calculation is shown in Figure 4 (for details see Table S3).

The *NetMHCIIpan* and TEPITOPE methods have similar predictive performance on the subset of 17 alleles covered by both methods. The TEPITOPE method has the highest performance for 10 alleles and the *NetMHCIIpan* the highest performance for 7 alleles (this difference is not significant *p*>0.3, Binomial test). For the 11 alleles *not* covered by the TEPITOPE method, *NetMHCIIpan* achieves the highest performance for 9 alleles, and the TEPITOPE method the highest performance for 2 alleles. For these alleles, *NetMHCIIpan* thus performs significantly better than the TEPITOPE method (*p*<0.01, Binominal test). Finally, for the 14 alleles not covered by the SMM-align method, and thus not included in the training of the pan-specific method, *NetMHCIIpan* achieves a higher performance than the TEPITOPE method. However, this difference is not significant. Also, in this experiment the *NetMHCIIpan* method performs particularly poorly compared to the TEPITOPE method on the DRB1*13 alleles. Using a network ensemble trained by leaving out the binding data for the DRB1*1302 allele, the average predictive performance for the DRB1*1302 allele is improved from 0.567 to 0.747 (data not shown). This result confirms our earlier observation that the DRB1*1302 allelic data included in the training of the *NetMHCIIpan* method forms an outlier group with unusual binding specificity characteristics.

### Identification of Peptide Binding Core

To validate the ability of the *NetMHCIIpan* method to correctly identify the binding core of peptides bound to MHC class II molecules, we compiled from the PDB database [34] a set of 15 peptides which have been crystallized in complex with an HLA-DR allele. For these peptides, we can identify the exact peptide binding by manual extracting which peptide residue is bound in the P1 pocket and subsequently test if this core can be identify by the prediction method. As demonstrated in Table 3, both the TEPITOPE and *NetMHCIIpan* methods are capable of identifying the binding core of the 15 peptides. TEPITOPE correctly identifies all 15 binding cores, whereas the *NetMHCIIpan* misaligns one peptide by a single amino acid residue.

**Table 3:** Identification of Peptide Binding Cores.

HLA-DRB	PDB ID	Length	Peptide sequence	Core	TEPITOPE	NetMHCIIpan
DRB1*0101	2FSE	14	AGFKGEQGPKGEPG	**FKGEQGPKG**	**FKGEQGPKG**	**FKGEQGPKG**
DRB1*0101	1KLG	15	GELIGILNAAKVPAD	**IGILNAAKV**	**IGILNAAKV**	**IGILNAAKV**
DRB1*0101	1SJE	16	PEVIPMFSALSEGATP	**VIPMFSALS**	**VIPMFSALS**	**VIPMFSALS**
DRB1*0101	1FYT	13	PKYVKQNTLKLAT	**YVKQNTLKL**	**YVKQNTLKL**	**YVKQNTLKL**
DRB1*0101	1AQD	15	VGSDWRFLRGYHQYA	**WRFLRGYHQ**	**WRFLRGYHQ**	**WRFLRGYHQ**
DRB1*0101	1PYW	11	XFVKQNAAALX	**FVKQNAAAL**	**FVKQNAAAL**	**FVKQNAAAL**
DRB1*0101	1T5X	15	AAYSDQATPLLLSPR	**YSDQATPLL**	**YSDQATPLL**	**YSDQATPLL**
DRB1*0301	1A6A	15	PVSKMRMATPLLMQA	**MRMATPLLM**	**MRMATPLLM**	**MRMATPLLM**
DRB1*0401	2SEB	12	AYMRADAAAGGA	**MRADAAAGG**	**MRADAAAGG**	**YMRADAAAG**
DRB1*0401	1J8H	13	PKYVKQNTLKLAT	**YVKQNTLKL**	**YVKQNTLKL**	**YVKQNTLKL**
DRB1*1501	1BX2	15	ENPVVHFFKNIVTPR	**VHFFKNIVT**	**VHFFKNIVT**	**VHFFKNIVT**
DRB1*1501	1YMM	23	ENPVVHFFKNIVTPRGGSGGGGG	**VHFFKNIVT**	**VHFFKNIVT**	**VHFFKNIVT**
DRB5*0101	1H15	14	GGVYHFVKKHVHES	**YHFVKKHVH**	**YHFVKKHVH**	**YHFVKKHVH**
DRB5*0101	1FV1	20	NPVVHFFKNIVTPRTPPPSQ	**FKNIVTPRT**	**FKNIVTPRT**	**FKNIVTPRT**
DRB5*0101	1ZGL	15	VHFFKNIVTPRTPGG	**FKNIVTPRT**	**FKNIVTPRT**	**FKNIVTPRT**

The table shows HLA-DR restricted peptides compiled from the PDB database [34]. The columns in the table give the HLA-DR restriction, the PDB identifier, and peptide length and peptide amino acid sequences, respectively. The last columns give the binding core as extracted from the protein complex crystal structure, and the core as predicted by the TEPITOPE and *NetMHCIIpan* methods, respectively.

### HLA-DR Allelic Specificity Clustering

**Figure 5:**
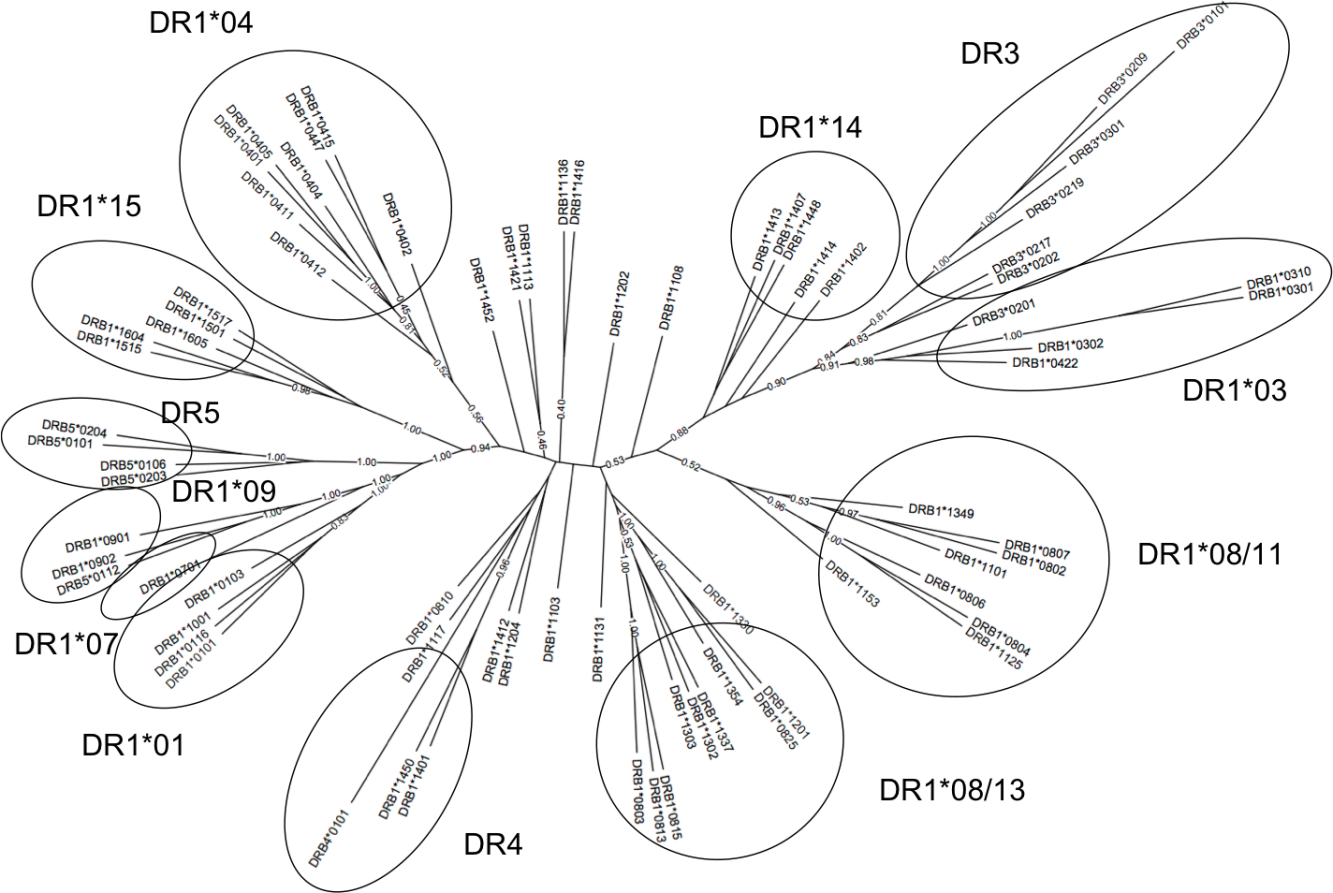
HLA-DR Clustering from *NetMHCIIpan* Predictions. The figure shows the clustering for 76 representative HLA-DR alleles. The tree was generated using the neighbor-joining algorithm from HLA distance matrices as described in the text. The circles are guides to the eye highlighting the suggested 12 HLA-DR supertypes.

It has previously been shown that HLA-A and HLA-B class I molecules can be clustered into a limited number of groups also known as supertypes sharing common binding specificity characteristics. A similar clustering of HLA-DR alleles has also been proposed [35]. In order to validate and extend this clustering, the *NetMHCIIpan* method was used to cluster HLA-DR molecules according to predicted peptide binding specificity. Pruned HLA distance trees were calculated as described in Materials and Methods. Figure 5 depicts a tree including 76 representatives of the currently known HLA-DR molecules.

The overall structure of the HLA-DR specificity tree is in accordance with the previously proposed clustering [35] containing 12 main supertypes. It is, however, striking to observe the high degree of serotype mixing between the different supertype clusters. Almost all of the proposed supertypes contain HLA-DR molecules from more than one serotype. This has earlier been observed when defining HLA-DR specific clusters based on the TEPITOPE binding matrices [35], but not to the degree suggested by the analysis presented here.

## Discussion

The MHC molecules are extremely polymorphic giving rise to many different peptide-binding specificities being expressed in the human population. More than 500 different HLA-DR molecules and more than 2000 different HLA-DQ and HLA-DP molecules have been described [2]. The only partially pan-specific HLA-DR prediction algorithm publicly available is the TEPITOPE method [27]. This method describes binding of peptides to 50 HLA-DR molecules. However, as shown in this work, the TEPITOPE method leaves large portions of the HLA-DR allelic polymorphism undescribed.

In the present work, we develop a HLA-DR pan-specific method, *NetMHCIIpan*, capable of providing quantitative predictions of peptide binding to all HLA-DR molecules with known protein sequence. The method is based on artificial neural networks and is trained on quantitative peptide HLA-DR binding data including the peptide-binding core, peptide flanking residues, and the HLA-DR residues estimated to be within interaction distance of the bound peptide. The natural strength of the method is the ability to predict binding of peptides to any HLA-DR molecule, thus being truly HLA-DR pan-specific. Further, since the method is artificial neural network based, it can capture non-linear relationships defining the binding specificity both within the peptide and between the peptide and the HLA molecule. This is fundamentally different from the methodology underlying the TEPITOPE method, that relies on the approximation that peptide binding specificities can be determined as a summation over independent HLA pockets preferences. The method is validated in terms of prediction of peptide binding to hitherto un-characterized HLA-DR molecules, large-scale leave-one-out experiments, cross-validation and identification of endogenously presented peptides and experimentally validated binding cores. In all validation experiments, the *NetMHCIIpan* method was shown to perform better than or comparable to TEPITOPE, the only other partially HLA-DR pan-specific binding prediction method publicly available.

A powerful application of the HLA-DR pan-specific prediction algorithm would be to search for highly promiscuous peptide sequences that will bind to most HLA-DR alleles. Such peptides could be of high value in the development of synthetic and recombinant vaccines, since they would bind universally in most humans independently of MHC class II genetic background and thus potentially provide universal helper T cell activation. By way of example, we applied the pan-specific method to identify peptides, predicted to bind a set of prevalent HLA-DR alleles. Prevalent alleles were selected as HLA-DR alleles with a maximal allelic frequency above 1% in an ethnic population as reported by Middleton et al. [36]. In doing so, we could identify peptides predicted to bind promiscuously to all prevalent HLA-DR molecules. Earlier efforts have been made to identify such highly promiscuous peptides. The PADRE sequence [37] is one of the most prominent examples of such peptides. Using the pan-specific method, the PADRE sequence is predicted to bind to less than 40% of the prevalent HLA-DR molecules. The analysis shown here demonstrates that exhaustive searches for truly pan-promiscuous HLA-DR are indeed feasible using the proposed pan-specific method.

The pan-specific approach relies on the ability of the neural networks to capture general features of the relationship between peptides and HLA sequences and interpret these in terms of a binding affinity. For this approach to provide reliable predictions, it is essential that polymorphism of the HLA molecules described by the pan-specific method is to some degree covered by the data included in the training of the method. For the *NetMHCIIpan* prediction method, we have included binding data covering only 14 of the more than 500 known HLA-DR molecules [2], thus very likely leaving large regions of the HLA specificity space uncovered. On the basis of the specificity clustering shown in Figure 5, we can identify HLA-DR alleles with un-characterized binding specificities as these alleles are found far from the alleles included in the training of the pan-specific method. Such novel HLA-DR molecules include the DRB1*14 molecules, i.e., DRB1*1407 (12.5%) and some of the DRB1*11, like DRB1*1103 (5%), as well as DRB1*12 alleles like DRB1*1202 (35%) placed close to center of the tree. The number in parenthesis after each allele is the maximal allelic frequency in an ethnic population as reported by Middleton et al. 2003 [36].

**Figure 6:**
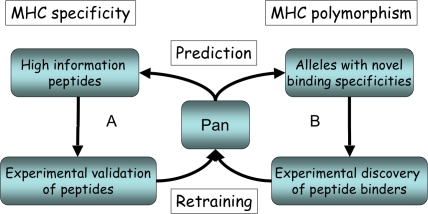
Strategy for Effective and Rational Coverage of the MHC Polymorphism and Specificity. (A) The pan-specific MHC class II prediction method is used to identify MHC alleles with novel binding specificities. These alleles have a predicted binding motif that is distant to all MHC class II molecules previously described. Subsequently, immunoassays are developed describing their binding specificity and data is fed back into a retraining of the pan-specific method. (B) Next, peptides with un-characterized binding affinity (high information peptides) are identifies, experimentally assayed and fed back into the retraining.

We have previously shown how integrative approaches combining bioinformatics and immunoassays to identify and experimental assay peptide with uncharacterized binding affinity can improve the prediction accuracy of peptide/MHC class I prediction algorithms [38]. Using the pan-specific approach to identify HLA class II molecules with uncharacterized binding specificities, we suggest extending this search strategy into the dimension of MHC polymorphism. A schematic illustration of this search strategy integrating bioinformatics and high throughput immunoassays is shown in Figure 6.

Here, we illustrate an iterative cycle that identifies novel MHC molecules with predicted binding specificities that are dissimilar to the specificities included in the training of the pan-specific method. Next, immunoassays should be developed describing the binding specificity of these molecules by identifying peptides with un-characterized binding affinity, and experimentally assay these peptides. Such an approach should allow for rapid and efficient sampling of both the MHC polymorphism and the diversity of peptide binding.

The current version of *NetMHCIIpan* and the benchmark data used in this work is available at http://www.cbs.dtu.dk/services/NetMHCIIpan. The service covers all HLA-DR alleles with known protein sequence. The method will be updated as more data becomes available. In the future, it is our hope to extend the method to also cover HLA-DQ and HLA-DP molecules.

## Materials and Methods

### Data

Quantitative HLA-DR restricted peptide-binding data was obtained from the IEDB database [28] and from an in-house collection of unpublished data [Bjorn Peters, private communication]. For external evaluation of the pan-specific method, we included a set of HLA-DR class II ligands from the SYFPEITHI database [29]. Only ligands not included in the quantitative HLA-DR restricted peptide binding data set were used. The SYFPEITHI data set consists of 584 MHC ligands restricted to 28 HLA-DR alleles. The details on the data set is given is Tables S4 and S5 (the complete data sets are available at http://www.cbs.dtu.dk/suppl/immunology/NetMHCIIpan.php).

### Method

The pan-specific HLA-DR method was constructed as described in Figure 1. The peptide nonamer core and peptide-flanking residues (PFR) were identified for each of the peptides in the IEDB dataset using the SMM-align method [26]. The SMM-align method identifies of the maximal scoring nonamer peptide core for each peptide sequence. This approach will thus leave out information on the suboptimal nonamer sequences that are predicted not to bind or to bind with a weaker affinity. To include information on the binding affinity for these suboptimal nonamer peptides, we assign a normalized binding score, *S*_norm_, to suboptimal nonamer peptides given as the ratio of the SMM-align score for the peptide to the SMM-align score of the optimal peptide multiplied with the log-transformed experimental IC50 binding value of the peptide. That is *S*_norm_ = (*S/S_M_*)*M*, where *S* is the SMM-align score for the (suboptimal) peptide, *S_M_* is the SMM-align score of the optimal peptide, and *M* is the binding value log-transformed as 1−log_50k_(aff), where aff is the experimental IC50 binding value of the full-length peptide, and log_50k_ is the logarithm with base 50.000. In case the SMM-align method assigns the maximal scoring nonamer peptide a log-transform binding value of 0, the log-transformed experimental IC50 binding value is assigned randomly to one of the suboptimal peptides and all other nonamer peptides are given a binding value of 0. In doing this expansion using sub-optimal nonamer peptides, the size of the IEDB dataset was enlarged from 14,607 to more than 100,000 data points. This more than 5 fold increase of the data gave consistent improvements to the accuracy of the prediction method in all benchmark calculations (data not shown).

For each peptide core, the PFRs were identified as the amino acids flanking the peptide core up to a maximum of three at either end.

### HLA Pseudo-Sequence

The HLA sequence was encoded in terms of a pseudo-sequence consisting of amino acid residues in contact with the peptide. The contact residues are defined as being within 4.0 Å of the peptide in any of a representative set of HLA class II structures. Only residues polymorphic in any known HLA-DR, DQ and DP protein sequence were included giving rise to a pseudo-sequence consisting of 21 amino acid residues. The HLA class II pseudo-sequence is described in detail in Table S6.

### Neural Network Training

Artificial neural networks (ANN) were trained to quantitatively predict peptide-HLA binding as described in Nielsen et al. [3]. The input sequences were presented to the neural network in three distinct manners: (a) conventional sparse encoding (i.e., encoded by 19 zeros and a one), (b) Blosum encoding, where each amino acid was encoded by the BLOSUM50 matrix score vector [39], and (c) a mixture of the two, where the peptide was sparse encoded and the HLA pseudo sequence was Blosum encoded. PFRs were calculated as the average BLOSUM62 score over a maximum length of three amino acids [26]. The PFR length was encoded as *L*_PFR_/3, 1−*L*_PFR_/3, where *L*_PFR_ is the length of the PFR (between 0 and 3), and the peptide length was encode as *L*_PEP_, 1−*L*_PEP_, where *L*_PEP_ = 1/(1+exp((*L*−15)/2)) and *L* is the peptide length. For each data point, the input to the neural network thus consists of the peptide sequence (9×20 = 180 inputs), the PFRs (2×20 = 40 inputs), the HLA pseudo sequence (21×20 = 420 inputs), the peptide length (2 inputs), and the length of the C and N terminal PFR's (2×2 = 4 inputs) resulting in a total of 646 input values.

To estimate the predictive performance of the method, the leave-one-out (LOO) experiment was conducted as described by Nielsen et al. [3]. For each HLA-DR molecule, a neural network ensemble was trained using all available data, excluding all data specific for the HLA-DR allele in question. Network architectures with hidden neurons of 22, 44, 56, and 66 were used. The network training was performed in a fivefold cross-validated manner using the three encoding schemes described above resulting in an ensemble of 60 neural networks (3 encoding schemes, 4 architectures, and 5 folds). The predicted affinity for a peptide was then determined as prediction value for the maximal scoring nonamer peptide core (including PFRs), where each nonamer peptide core is scored as the average of the 60 predictions in the neural network ensemble.

For the final *NetMHCIIpan* method, a conventional five-fold cross-validated training was performed. The pool of unique peptides was randomly split into five groups with all HLA binding data for a given peptide placed in the same group (in this way, no peptide can belong to more than one group).

### Nearest Neighbor Distance

The nearest neighbor distance between two HLA alleles is estimated from the alignment score of the HLA pseudo sequences using the relation *d* = 1−*s*(*A*,*B*)/(*s*(*A*,*A*)*s*(*B*,*B*))^1/2^, where *s*(*A*,*B*) is the BLOSUM50 alignment score [39] between the pseudo sequences *A* and *B*, respectively.

### HLA Distance Trees

HLA distance trees were derived from correlations between predicted binding affinities as described by Nielsen et al. [3]. In order to visualize the HLA distance tree, only a subset of the leaves in the tree was displayed. The subset was selected in a Hobohm 1-like manner, where the alleles were clustered at a 0.95 distance level and only a single allele from each cluster selected for display [40].

### In Vitro Binding Assay

The extracellular parts of HLA DRA1*0101 and HLA DRB1*0813 were fused to the Fos Jun leucine zipper dimerization motifs as previously described [41]. Both chains were separately expressed as inclusion bodies in *E. coli* (BL21) using standard IPTG induction. The two chains were extracted from inclusion bodies and purified by anion exchange and gel filtration chromatography under denaturing conditions. Equimolar concentrations of alpha and beta chain were diluted into a refolding buffer containing a titration of peptide (0–15 µM). After 48 h of incubation at 18°C the concentration of formed complex was determined by a quantitative ELISA using the HLA-DR specific monoclonal antibody L243. The data was fitted to a saturation curve using non-linear regression and the *K*_d_ value determined.

## Supporting Information

Table S1 Leave-One-Molecule Out (LOO) Benchmark Results in Terms of the Spearman's Rank Correlation. The table gives the allele name, the number of peptide included in the IEDB data for each allele, the LOO performance, the nearest neighbor SMM-align [26] performance together with the distance to that neighbor and the neighbor allele name and the performance of the TEPITOPE method [25],[27] for the subset of alleles covered by that method. The Ave* row give the average performance over all 14 alleles, and the Ave** row gives the average performance over the 11 alleles covered by the TEPITOPE method.
(0.08 MB DOC)

Table S2 Cross-Validated Benchmark Calculation. The predictive performance between the pan-specific, SMM-align, and TEPITOPE methods compared in terms of the AUC value and Pearson's correlation. The first column gives the allele name, the second column gives the number of data included for each allele, the third and fourth columns give the predictive performance for the pan-specific method, the sixth and seventh columns the predictive performance for the SMM-align method, and the last column the predictive performance for the TEPITOPE method. The Ave* row give the average performance over all 14 alleles, and the Ave** row gives the average performance over the 11 alleles covered by the TEPITOPE method.
(0.08 MB DOC)

Table S3 Prediction of Endogenously Presented Peptides. The benchmark data set consists of 584 HLA-DR restricted ligands covering 28 HLA-DR alleles downloaded from the SYFPEITHI database as described in the text. The table gives the allele name, the number of HLA ligands restricted to each allele, and the average AUC values for the ligands restricted to each allele for the NetMHCIIpan (PAN), and TEPITOPE methods, respectively. The last two columns indicate if the allele is covered (v) by the SMM-align (in PAN) and TEPITOPE (in TEPITOPE) methods, respectively, or not. If the allele is not covered by the TEPITOPE method, the closest allele covered by the TEPITOPE method as identified by sequence similarity between the HLA pseudo-sequences is used. Ave* and Ave** give the average performance over all 28 alleles and the 17 alleles covered by the TEPITOPE method, respectively. Ave*** gives the average performance over the 11 alleles not covered by the TEPITOPE method, and Ave**** gives the average performance for the 14 alleles not covered by the SMM-align method.
(0.08 MB DOC)

Table S4 IEDB Quantitative HLA-DR Restricted Peptide Binding Data. 14 HLA-DR alleles are covered by the data set. The first column gives the HLA-DR allele, the second column the number of peptide data for each allele, and the third and fourth columns give the number of peptide binders/non-binders, respectively. Peptide binders are classified using an IC50 threshold value of 500 nM.
(0.05 MB DOC)

Table S5 The SYFPEITHI Data Set. HLA-DR ligands downloaded from the SYFPEITHI database [29]. The first and third columns give the allele names, and the second and fourth column give the number of HLA-DR ligands for each allele.
(0.05 MB DOC)

Table S6 The HLA Class II Pseudo-Sequence. The table shows the HLA class II pseudo-sequence. The columns gives the pseudo sequence position, the HLA residue numbering according to the IMGT nomenclature [2], and the amino acid polymorphism at each position in the pseudo sequence for known HLA-DR, DP and DQ loci protein sequences (as of November 2007). Note, that the DPB protein sequence has a deletion of two amino acids at position 24–25 compared to DRB. The DQB sequence numbering for DPB after position 25 is off by two. For DPB position 26 thus corresponds to position 24 in the DPB protein sequence.
(0.09 MB DOC)
