# Supplementary material for: Quantitative Predictions of Peptide Binding to Any HLA-DR Molecule of Known Sequence: NetMHCIIpan
Source: PLoS Comput Biol. 2008 Jul 4;4(7):e1000107. doi: 10.1371/journal.pcbi.1000107 (PMC2430535; doi:10.1371/journal.pcbi.1000107)
Supplement: Table S1 — Leave-One-Molecule Out (LOO) Benchmark Results in Terms of the Spearman's Rank Correlation. The table gives the allele name, the number of peptide included in the IEDB data for each allele, the LOO performance, the nearest neighbor SMM-align [26] performance together with the distance to that neighbor and the neighbor allele name and the performance of the TEPITOPE method [25],[27] for the subset of alleles covered by that method. The Ave* row give the average performance over all 14 alleles, and the Ave** row gives the average performance over the 11 alleles covered by the TEPITOPE method. (0.08 MB DOC) [file pcbi.1000107.s001.doc]

**Supplementary Table 1. Leave-one-molecule out (LOO) benchmark results in terms of the Spearman’s rank correlation.**

| **Spearman’s Rank correlation** | | | | | | |
| --- | --- | --- | --- | --- | --- | --- |
| **Allele** | **N** | **LOO** | **Neighbor** | **Dist** | **Neighbor allele** | **TEPITOPE** |
| DRB1*0101 | 5166 | 0.570 | 0.487 | 0.352 | DRB1*0401 | 0.479 |
| DRB1*0301 | 1020 | 0.449 | 0.343 | 0.277 | DRB3*0101 | 0.260 |
| DRB1*0401 | 1024 | 0.598 | 0.503 | 0.066 | DRB1*0405 | 0.450 |
| DRB1*0404 | 663 | 0.684 | 0.592 | 0.091 | DRB1*0401 | 0.525 |
| DRB1*0405 | 630 | 0.597 | 0.550 | 0.066 | DRB1*0401 | 0.513 |
| DRB1*0701 | 853 | 0.655 | 0.538 | 0.504 | DRB1*0901 | 0.526 |
| DRB1*0802 | 420 | 0.631 | 0.590 | 0.111 | DRB1*1101 | 0.460 |
| DRB1*0901 | 530 | 0.388 | 0.340 | 0.431 | DRB5*0101 |  |
| DRB1*1101 | 950 | 0.588 | 0.413 | 0.084 | DRB1*1302 | 0.458 |
| DRB1*1302 | 498 | 0.351 | 0.343 | 0.084 | DRB1*1101 | 0.324 |
| DRB1*1501 | 934 | 0.535 | 0.482 | 0.295 | DRB1*0404 | 0.429 |
| DRB3*0101 | 549 | 0.444 | 0.381 | 0.277 | DRB1*0301 |  |
| DRB4*0101 | 446 | 0.469 | 0.443 | 0.397 | DRB1*0404 |  |
| DRB5*0101 | 924 | 0.633 | 0.599 | 0.295 | DRB1*1101 | 0.412 |
| **Ave*** |  | 0.542 | 0.472 |  |  |  |
| **Ave**** |  | 0.572 | 0.495 |  |  | 0.440 |

The table gives the allele name, the number of peptide included in the IEDB data for each allele, the LOO performance, the nearest neighbor SMM-align [26] performance together with the distance to that neighbor and the neighbor allele name and the performance of the TEPITOPE method [25,27] for the subset of alleles covered by that method. The Ave* row give the average performance over all 14 alleles, and the Ave** row gives the average performance over the 11 alleles covered by the TEPITOPE method.
