# Supplementary material for: Quantitative Predictions of Peptide Binding to Any HLA-DR Molecule of Known Sequence: NetMHCIIpan
Source: PLoS Comput Biol. 2008 Jul 4;4(7):e1000107. doi: 10.1371/journal.pcbi.1000107 (PMC2430535; doi:10.1371/journal.pcbi.1000107)
Supplement: Table S2 — Cross-Validated Benchmark Calculation. The predictive performance between the pan-specific, SMM-align, and TEPITOPE methods compared in terms of the AUC value and Pearson's correlation. The first column gives the allele name, the second column gives the number of data included for each allele, the third and fourth columns give the predictive performance for the pan-specific method, the sixth and seventh columns the predictive performance for the SMM-align method, and the last column the predictive performance for the TEPITOPE method. The Ave* row give the average performance over all 14 alleles, and the Ave** row gives the average performance over the 11 alleles covered by the TEPITOPE method. (0.08 MB DOC) [file pcbi.1000107.s002.doc]

**Supplementary Table 2. Cross-validated benchmark calculation.**

|  |  | **Pan** | | **SMM-align** | | **TEPITOPE** |
| --- | --- | --- | --- | --- | --- | --- |
| **Allele** | **N** | **Pearson** | **AUC** | **Pearson** | **AUC** | **AUC** |
| DRB1*0101 | 5166 | 0.681 | 0.840 | 0.610 | 0.802 | 0.720 |
| DRB1*0301 | 1020 | 0.670 | 0.846 | 0.563 | 0.795 | 0.664 |
| DRB1*0401 | 1024 | 0.627 | 0.815 | 0.496 | 0.751 | 0.716 |
| DRB1*0404 | 663 | 0.701 | 0.858 | 0.579 | 0.801 | 0.770 |
| DRB1*0405 | 630 | 0.637 | 0.833 | 0.560 | 0.789 | 0.759 |
| DRB1*0701 | 853 | 0.728 | 0.869 | 0.618 | 0.812 | 0.761 |
| DRB1*0802 | 420 | 0.684 | 0.856 | 0.555 | 0.787 | 0.766 |
| DRB1*0901 | 530 | 0.525 | 0.731 | 0.360 | 0.655 |  |
| DRB1*1101 | 950 | 0.723 | 0.870 | 0.581 | 0.796 | 0.721 |
| DRB1*1302 | 498 | 0.674 | 0.824 | 0.558 | 0.785 | 0.652 |
| DRB1*1501 | 934 | 0.643 | 0.802 | 0.528 | 0.727 | 0.686 |
| DRB3*0101 | 549 | 0.633 | 0.844 | 0.585 | 0.836 |  |
| DRB4*0101 | 446 | 0.695 | 0.876 | 0.541 | 0.793 |  |
| DRB5*0101 | 924 | 0.716 | 0.863 | 0.529 | 0.761 | 0.680 |
| **Ave*** | **14** | **0.667** | **0.838** | **0.547** | **0.778** |  |
| **Ave**** | **11** | **0.680** | **0.843** | **0.562** | **0.782** | **0.718** |

The predictive performance between the pan-specific, SMM-align, and TEPITOPE methods compared in terms of the AUC value and Pearson’s correlation. The first column gives the allele name, the second column gives the number of data included for each allele, the third and fourth columns give the predictive performance for the pan-specific method, the sixth and seventh columns the predictive performance for the SMM-align method, and the last column the predictive performance for the TEPITOPE method. The Ave* row give the average performance over all 14 alleles, and the Ave** row gives the average performance over the 11 alleles covered by the TEPITOPE method.
