# Supplementary material for: Quantitative Predictions of Peptide Binding to Any HLA-DR Molecule of Known Sequence: NetMHCIIpan
Source: PLoS Comput Biol. 2008 Jul 4;4(7):e1000107. doi: 10.1371/journal.pcbi.1000107 (PMC2430535; doi:10.1371/journal.pcbi.1000107)
Supplement: Table S3 — Prediction of Endogenously Presented Peptides. The benchmark data set consists of 584 HLA-DR restricted ligands covering 28 HLA-DR alleles downloaded from the SYFPEITHI database as described in the text. The table gives the allele name, the number of HLA ligands restricted to each allele, and the average AUC values for the ligands restricted to each allele for the NetMHCIIpan (PAN), and TEPITOPE methods, respectively. The last two columns indicate if the allele is covered (v) by the SMM-align (in PAN) and TEPITOPE (in TEPITOPE) methods, respectively, or not. If the allele is not covered by the TEPITOPE method, the closest allele covered by the TEPITOPE method as identified by sequence similarity between the HLA pseudo-sequences is used. Ave* and Ave** give the average performance over all 28 alleles and the 17 alleles covered by the TEPITOPE method, respectively. Ave*** gives the average performance over the 11 alleles not covered by the TEPITOPE method, and Ave**** gives the average performance for the 14 alleles not covered by the SMM-align method. (0.08 MB DOC) [file pcbi.1000107.s003.doc]

**Supplementary Table 3. Prediction of endogenously presented peptides.**

| **Allele** | **N** | **PAN** | **TEPITOPE** | **In PAN** | **In TEPITOPE** |
| --- | --- | --- | --- | --- | --- |
| DRB1*0101 | 35 | 0.874 | **0.880** | v | v |
| DRB1*0102 | 6 | **0.897** | 0.895 |  | v |
| DRB1*0301 | 26 | **0.882** | 0.837 | v | v |
| DRB1*0401 | 202 | 0.865 | **0.880** | v | v |
| DRB1*0402 | 34 | 0.722 | **0.888** |  | v |
| DRB1*0403 | 1 | **0.991** | 0.954 |  |  |
| DRB1*0404 | 42 | **0.857** | 0.829 | v | v |
| DRB1*0405 | 35 | **0.848** | 0.809 | v | v |
| DRB1*0701 | 35 | 0.678 | **0.704** | v | v |
| DRB1*0801 | 36 | 0.645 | **0.694** |  | v |
| DRB1*0802 | 1 | **0.982** | 0.914 | v | v |
| DRB1*0803 | 1 | **0.659** | 0.065 |  |  |
| DRB1*0901 | 4 | **0.866** | 0.642 | v |  |
| DRB1*1001 | 1 | 0.966 | **0.980** |  |  |
| DRB1*1101 | 28 | 0.860 | **0.870** | v | v |
| DRB1*1104 | 8 | **0.892** | 0.836 |  | v |
| DRB1*1201 | 7 | **0.799** | 0.693 |  |  |
| DRB1*1301 | 20 | 0.721 | **0.854** |  | v |
| DRB1*1302 | 17 | 0.567 | **0.726** | v | v |
| DRB1*1401 | 5 | **0.636** | 0.495 |  |  |
| DRB1*1501 | 10 | **0.839** | 0.830 | v | v |
| DRB1*1502 | 3 | 0.569 | **0.768** |  | v |
| DRB3*0101 | 2 | **0.957** | 0.888 | v |  |
| DRB3*0202 | 3 | **0.727** | 0.569 |  |  |
| DRB3*0301 | 5 | **0.816** | 0.675 |  |  |
| DRB4*0101 | 4 | 0.471 | **0.567** | v |  |
| DRB4*0103 | 1 | **0.929** | 0.910 |  |  |
| DRB5*0101 | 12 | 0.930 | **0.934** | v | v |
| **Ave*** | **28** | 0.802 | 0.771 | Alleles | |
| **Ave**** | **17** | 0.802 | 0.832 | TEPITOPE alleles | |
| **Ave***** | **11** | 0.802 | 0.676 | Non TEPITOPE alleles | |
| **Ave****** | **14** | 0.784 | 0.734 | Non SMM-align alleles | |

The benchmark data set consists of 584 HLA-DR restricted ligands covering 28 HLA-DR alleles downloaded from the SYFPEITHI database as described in the text. The table gives the allele name, the number of HLA ligands restricted to each allele, and the average AUC values for the ligands restricted to each allele for the *NetMHCIIpan* (PAN), and TEPITOPE methods, respectively. The last two columns indicate if the allele is covered (v) by the SMM-align (in PAN) and TEPITOPE (in TEPITOPE) methods, respectively, or not. If the allele is not covered by the TEPITOPE method, the closest allele covered by the TEPITOPE method as identified by sequence similarity between the HLA pseudo-sequences is used. Ave* and Ave** give the average performance over all 28 alleles and the 17 alleles covered by the TEPITOPE method, respectively. Ave*** gives the average performance over the 11 alleles *no*t covered by the TEPITOPE method, and Ave**** gives the average performance for the 14 allele not covered by the SMM-align method.
