# Supplementary material for: Quantitative Predictions of Peptide Binding to Any HLA-DR Molecule of Known Sequence: NetMHCIIpan
Source: PLoS Comput Biol. 2008 Jul 4;4(7):e1000107. doi: 10.1371/journal.pcbi.1000107 (PMC2430535; doi:10.1371/journal.pcbi.1000107)
Supplement: Table S4 — IEDB Quantitative HLA-DR Restricted Peptide Binding Data. 14 HLA-DR alleles are covered by the data set. The first column gives the HLA-DR allele, the second column the number of peptide data for each allele, and the third and fourth columns give the number of peptide binders/non-binders, respectively. Peptide binders are classified using an IC50 threshold value of 500 nM. (0.05 MB DOC) [file pcbi.1000107.s004.doc]

Supplementary Table 4. IEDB quantitative HLA-DR restricted peptide binding data.

| Allele | N | # Binders | # Non-binders |
| --- | --- | --- | --- |
| DRB1*0101 | 5166 | 3510 | 1656 |
| DRB1*0301 | 1020 | 277 | 743 |
| DRB1*0401 | 1024 | 510 | 514 |
| DRB1*0404 | 663 | 386 | 277 |
| DRB1*0405 | 630 | 425 | 205 |
| DRB1*0701 | 853 | 498 | 355 |
| DRB1*0802 | 420 | 148 | 272 |
| DRB1*0901 | 530 | 254 | 276 |
| DRB1*1101 | 950 | 429 | 521 |
| DRB1*1302 | 498 | 199 | 299 |
| DRB1*1501 | 934 | 450 | 484 |
| DRB3*0101 | 549 | 75 | 474 |
| DRB4*0101 | 446 | 200 | 246 |
| DRB5*0101 | 924 | 478 | 446 |
| Total | 14607 | 7839 | 6768 |

14 HLA-DR alleles are covered by the data set. The first column gives the HLA-DR allele, the second column the number of peptide data for each allele, and the third and fourth columns give the number of peptide binders/non-binders, respectively. Peptide binders are classified using an IC50 threshold value of 500 nM.
