# Supplementary material for: Quantitative Predictions of Peptide Binding to Any HLA-DR Molecule of Known Sequence: NetMHCIIpan
Source: PLoS Comput Biol. 2008 Jul 4;4(7):e1000107. doi: 10.1371/journal.pcbi.1000107 (PMC2430535; doi:10.1371/journal.pcbi.1000107)
Supplement: Table S5 — The SYFPEITHI Data Set. HLA-DR ligands downloaded from the SYFPEITHI database [29]. The first and third columns give the allele names, and the second and fourth column give the number of HLA-DR ligands for each allele. (0.05 MB DOC) [file pcbi.1000107.s005.doc]

Supplementary Table 5. The SYFPEITHI data set.

| Allele | # | Allele | # |
| --- | --- | --- | --- |
| DRB1*0101 | 35 | DRB1*1101 | 28 |
| DRB1*0102 | 6 | DRB1*1104 | 8 |
| DRB1*0301 | 26 | DRB1*1201 | 7 |
| DRB1*0401 | 202 | DRB1*1301 | 20 |
| DRB1*0402 | 34 | DRB1*1302 | 17 |
| DRB1*0403 | 1 | DRB1*1401 | 5 |
| DRB1*0404 | 42 | DRB1*1501 | 10 |
| DRB1*0405 | 35 | DRB1*1502 | 3 |
| DRB1*0701 | 35 | DRB3*0101 | 2 |
| DRB1*0801 | 36 | DRB3*0202 | 3 |
| DRB1*0802 | 1 | DRB3*0301 | 5 |
| DRB1*0803 | 1 | DRB4*0101 | 4 |
| DRB1*0901 | 4 | DRB4*0103 | 1 |
| DRB1*1001 | 1 | DRB5*0101 | 12 |
|  |  | Total | 584 |

### HLA-DR ligands downloaded from the SYFPEITHI database [29]. The first and third columns give the allele names, and the second and fourth column give the number of HLA-DR ligands for each allele.
