# Supplementary material for: Quantitative Predictions of Peptide Binding to Any HLA-DR Molecule of Known Sequence: NetMHCIIpan
Source: PLoS Comput Biol. 2008 Jul 4;4(7):e1000107. doi: 10.1371/journal.pcbi.1000107 (PMC2430535; doi:10.1371/journal.pcbi.1000107)
Supplement: Table S6 — The HLA Class II Pseudo-Sequence. The table shows the HLA class II pseudo-sequence. The columns gives the pseudo sequence position, the HLA residue numbering according to the IMGT nomenclature [2], and the amino acid polymorphism at each position in the pseudo sequence for known HLA-DR, DP and DQ loci protein sequences (as of November 2007). Note, that the DPB protein sequence has a deletion of two amino acids at position 24–25 compared to DRB. The DQB sequence numbering for DPB after position 25 is off by two. For DPB position 26 thus corresponds to position 24 in the DPB protein sequence. (0.09 MB DOC) [file pcbi.1000107.s006.doc]

**Supplementary Table 6. The HLA class II pseudo sequence.**

| Pseudo sequence  Position | Protein  position | DR | DP^§^ | DQ | All |
| --- | --- | --- | --- | --- | --- |
| 1 | 9 | WEKQ | YFHD | YFL | WEKQYFHDL |
| 2 | 11 | LSIVGDPRA | GL | F | LSIVGDPRAF |
| 3 | 13 | FSHPYGRC | Q | GA | FSHPYGRCQA |
| 4 | 14 | EK | E | LM | EKLM |
| 5 | 26 | LFYN | F | GLY | LFYNG |
| 6 | 28 | EDHIN | E | TS | EDHITSN |
| 7 | 30 | CLYHGRDF | YD | HYS | CLYHGRDSF |
| 8 | 47 | YFL | F | YF | YFL |
| 9 | 56 | PA | P | PL | PAL |
| 10 | 57 | DASVIT | AED | VSDA | DASVIET |
| 11 | 60 | YHSN | Y | YN | YHSN |
| 12 | 67 | LIF | NILF | VID | LIFVDN |
| 13 | 70 | QDRE | E | GER | QDRGE |
| 14 | 71 | REAKT | REK | ADKT | REAKDT |
| 15 | 74 | AREQLV | LV | SAE | AREQLSV |
| 16 | 77 | TNA | R | RT | TNRA |
| 17 | 78 | YHV | IMV | V | YHVR |
| 18 | 81 | HY | H | H | HY |
| 19 | 85 | VA | L | VL | VAEGL |
| 20 | 86 | GVAD | DGV | AE | GVAED |
| 21 | 89 | F | MV | GT | FLGT |

The table shows the HLA class II pseudo sequence. The columns gives the pseudo sequence position, the HLA residue numbering according to the IMGT nomenclature [2], and the amino acid polymorphism at each position in the pseudo sequence for known HLA-DR, DP and DQ loci protein sequences (as of November 2007). ^§^Note, that the DPB protein sequence has a deletion of two amino acids at position 24-25 compared to DRB. The DQB sequence numbering for DPB after position 25 is off by two. For DPB position 26 thus corresponds to position 24 in the DPB protein sequence.
